# Magnetic Graphene Field-Effect Transistor Biosensor for Single-Strand DNA Detection

**DOI:** 10.1186/s11671-019-3048-1

**Published:** 2019-07-24

**Authors:** Jinjin Sun, Xiaohui Xie, Ke Xie, Shicai Xu, Shouzhen Jiang, Junfeng Ren, Yuefeng Zhao, Huaqiang Xu, Jingjing Wang, Weiwei Yue

**Affiliations:** 1 Shandong Province Key Laboratory of Medical Physics and Image Processing Technology, School of Physics and Electronics, Shandong Normal University, Jinan, 250358 People’s Republic of China; 2 Institute of Materials and Clean Energy, Shandong Normal University, Jinan, 250358 People’s Republic of China; 3 College of Physics and Electronics, Dezhou University, Dezhou, 253000 People’s Republic of China

**Keywords:** Magnetic, Graphene, Field-effect transistor, Biosensor, Magnetic nanobeads, DNA

## Abstract

Herein, a magnetic graphene field-effect transistor biosensor was prepared through the transfer of a chemical vapor deposition graphene film onto a glass substrate to produce a sensing film and conductive channel. By fixing 1-pyrenebutanoic acid succinimidyl ester onto graphene film as an anchor, a probe aptamer was immobilized on the graphene film in order to capture magnetically labeled complementary single-stranded DNA. Our experiments showed that, within a periodic magnetic field, the biosensor impedance exhibited a periodic oscillation, the amplitude of which was correlated to the complementary DNA concentration. Based on this principle, the magnetic graphene field-effect transistor was utilized to detect single-stranded DNA with detection limition of 1 pM. The results were rationalized using a model wherein the magnetic force causes the DNA strand to bend, thereby resulting in magnetic nanobeads/DNA modulation of the double conductive layer of graphene transistors. Furthermore, since a periodic magnetic field could be introduced to produce a periodic impedance changes of MGFETs, sampling integration could be used to improve the signal-to-noise ratio efficiently by increasing the number of periods of the external magnetic field. Therefore, a novel biosensor for DNA detection with high sensitivity has been presented in this work. Based on the detection principle, this system may also be a potential tool for detecting other bio-molecules, cells, etc.

## Introduction

The detection of DNA is of great significance for the study of molecular biology and the diagnosis of genetic diseases [1–3]. To date, various biosensors for DNA detection have been developed, including fluorescent biosensors [4, 5], electrochemical biosensors [6–9], and field-effect transistor (FET) biosensors [10–13], with the latter having attracted widespread attention due to their high sensitivity and specificity. Kaisti et al. [12] developed a FET biosensor to detect unlabeled single-stranded DNA using peptide nucleic acid probes. Kim et al. [13] fabricated a FET-type DNA charge sensor based on standard complementary metal oxide semiconductor technology.

Due to its high specific surface area, high electrical conductivity, and excellent electron mobility, graphene has been heralded an ideal material for the fabrication of FET biosensors [14–16]. Cai et al. [15] developed a graphene FET (GFET) biosensor for ultrasensitive detection of DNA via peptide nucleic acid DNA hybridization. Our group has also proposed a multi-channel GFET biosensor to determine the binding kinetics and affinity of DNA hybridization and single-base mismatched [16].

In a conventional GFET, an external gate electrode electric field generates a double conductive layer at the interface between the graphene film and the solution electrolyte [17–19]. Based on a captive model of GFETs [16], the gate electrode charges and discharges the double conductive layer through the electrolyte, thereby modulating the GFET conductivity. Therefore, the conductivity of a GFET is related to the intensity of the external electric field and the ion concentration in electrolyte.

During the research, it was found that the research on the sensitivity of GFETs has reached the fM level. For example, Ping et al. [20] and Zheng et al. [21] have reported conventional GFET biosensors with detection limit in fM level. However, the above literature achieves extremely high sensitivity by semiconductor analyzer detection, which is expensive and inconvenient for practical applications. Furthermore, Ag/AgCl electrodes are commonly used as external gate electrodes, which are unsuitable for the construction of integrated biosensors due to their size and reusability.

Herein, a magnetic GFET (MGFET) biosensor, in which a magnetic field rather than an electrical field is utilized to modulate the GFET conductivity, was developed. The conductive channel was achieved using a chemical vapor deposition (CVD) graphene film transferred onto a glass substrate with two indium tin oxide (ITO) electrodes. The graphene film was functionalized with 1-pyrenebutanoic acid succinimidyl ester (PBASE) to allow linkage of a probe aptamer to capture and hybridize with complementary magnetically labeled single-stranded DNA (cDNA). Applying a periodic magnetic field on the back side of the MGFETs, a periodic MGFET electric impedance was achieved. Further, the electric impedance fluctuation of the MGFETs in a periodic magnetic field was related to the concentration of cDNA. A corresponding lab-made detection device was constructed to detect MGFET impedance in real time. Since the magnetic field is not in contact with the MGFETs directly, the MGFETs prepared herein are easier to integrate and apply than conventional GFET biosensors. The preparation of MGFETs, the construction of the lab-made detection system, and the detection principle were all described in detail in this paper.

## Methods

### Materials and Instrument

A glass substrate with ITO electrodes was purchased from Hua Nan Xiang Cheng Ltd. (China). The probe aptamer, cDNA, and mismatched DNA were purchased from Sangon Biotech Inc. (Shanghai, China). The sequence of the probe aptamer was (5′-NH_2_-TGG ACC CCC TCA TAA CGC CTC CTT TTC-FAM-3′), sequence of the complementary DNA was (5′-NH_2_-GAA AAG GAG GCG TTA TGA GGG GGT CCA-3′), sequence of the completely mismatched DNA was (5′-NH_2_-TCC CCT TCT TAT GGC CTG TTT TTC AAC-3′), and sequence of the single-base mismatched DNA was (5′-NH_2_-GAA AAG GAG TCG TTA TGA GGG GGT CCA-3′). PBASE and dimethyl sulfoxide (DMSO) were obtained from Sigma-Aldrich (Shanghai, China). Magnetic nanobeads (MBs) modified with carboxyl groups (10 mg/mL) were obtained from Xianfeng Nano Material Technology Co., Ltd. (Nanjing, China). 1-Ethyl-3-(3-dimethylaminopropyl) carbodiimide hydrochloride, N-hydroxysuccinimide, sodium dodecylbenzenesulfonate (SDS), and sodium dodecyl sulfate phosphate-buffered saline (PBS, P5368-10PAK; pH 7.4) were purchased from Sigma-Aldrich (Shanghai, China).

A Raman microscopic system (SPEX-1403, SPEX) was used to characterize the quality of graphene as well as to verify the functionalization of MGFETs. A fluorescence photometer (LS55, PerkinElmer) was used to characterize the coupling of magnetic nanoparticles to cDNA. A lab-made data acquisition system was used to record the impedance of MGFETs in real time.

### Coupling cDNA to MBs

After uniformly dispersed through ultrasound for 20 min, a 20 μL suspension of MBs modified with carboxyl groups was mixed with 200 μL of 1-ethyl-3-(3-dimethylaminopropyl) carbodiimide hydrochloride (2 mg/mL) and 200 μL of N-hydroxysuccinimide (2 mg/mL) for 15 min to obtain activated MBs [22, 23]. Then, 20 μL of cDNA solution was added to the MBs solution and incubated for 2 h at room temperature with continuous gentle shaking. A magnetic field was then introduced to enrich the cDNA samples through MBs. The magnetic nanobeads/DNA (MB/cDNA) conjugates were washed three times with PBS and dispersed in PBS for future use.

### Fabrication of MGFETs

**Fig. 1:**
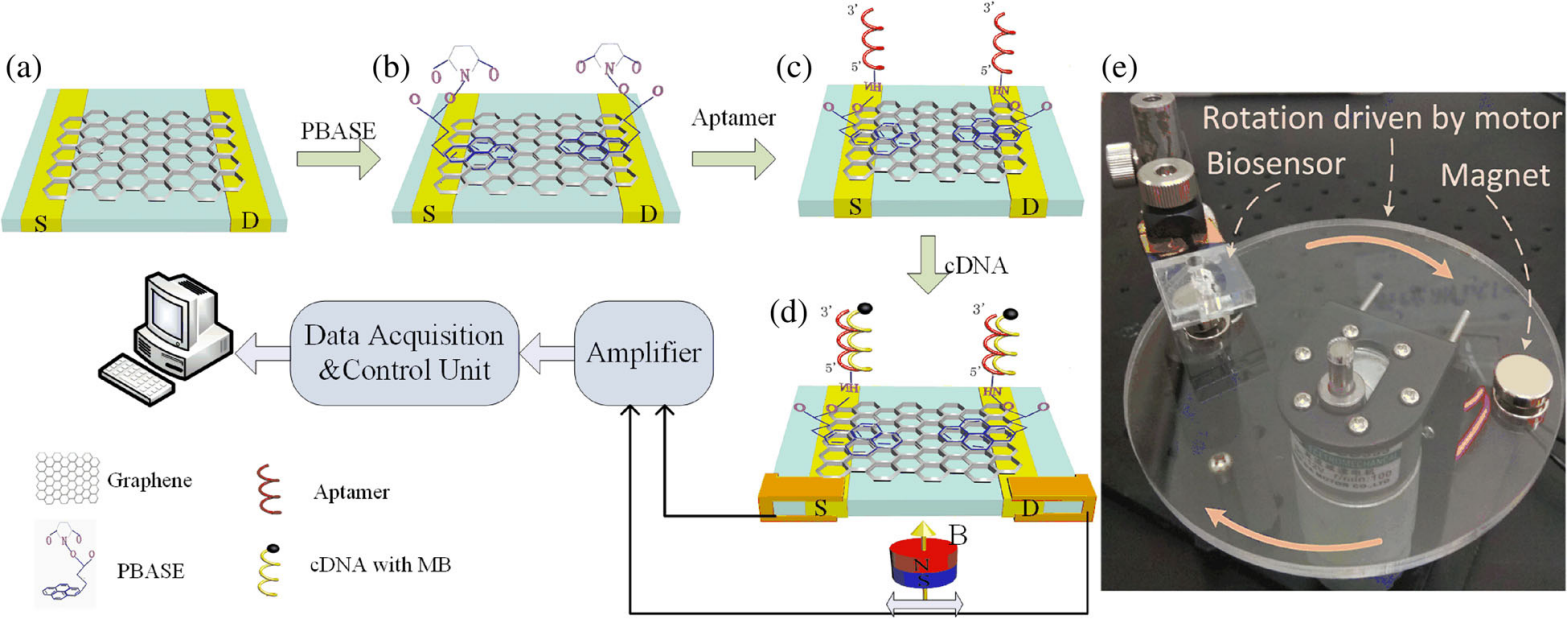
Functionalization and detection principle of the MGFETs. **a** Graphene film grown by chemical vapor deposition. **b** Functionalization of graphene by PBASE. **c** Immobilization of probe aptamer via PBASE. **d** Hybridization of the probe aptamer with cDNA. **e** Photograph of the detection device

The preparation of MGFETs is described in detail below. Firstly, a CVD graphene film was transferred onto a glass plate as the conductive channel between the two ITO electrodes (Fig. 1a), as described previously [18, 19]. Secondly, PBASE (10 mM) dissolved in DMSO was injected into the MGFETs for 12 h at room temperature and allowed to react completely with graphene through π–π stacking (Fig. 1b). The MGFETs were then washed successively with DMSO and PBS to remove any unreacted PBASE. Thirdly, 2 μM of the probe aptamer was introduced into the MGFETs and incubated with PBASE for 4 h at room temperature, allowing the probe aptamer to react sufficiently with PBASE (Fig. 1c). The MGFETs were then respectively washed with 0.2% SDS three times to remove any unbound probe aptamer.

## Results and Discussion

### Characterization of MGFETs

**Fig. 2:**
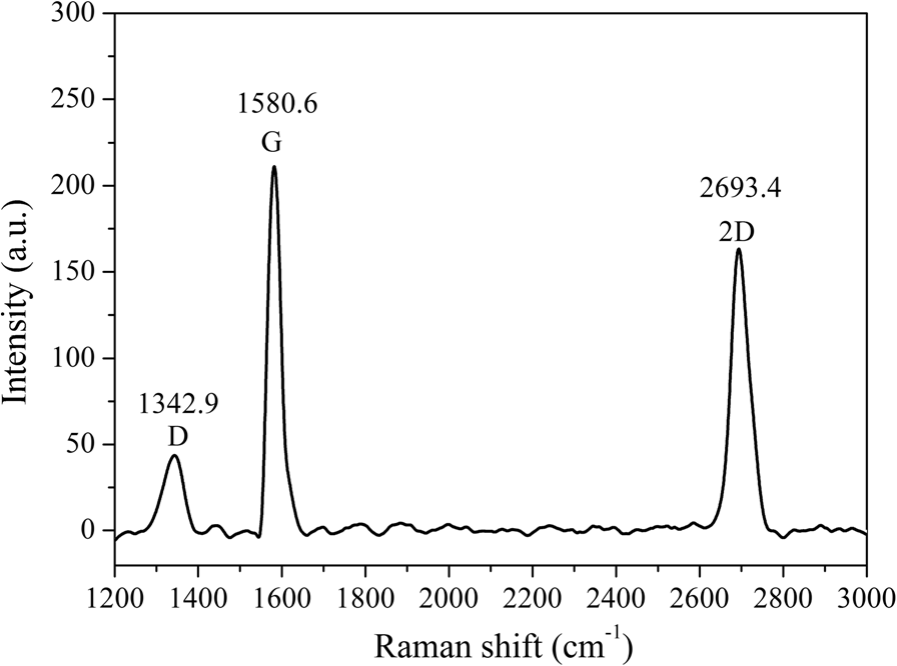
Raman spectrum

Graphene film produced by the CVD method was transferred on a glass substrate as a conductive channel between two ITO electrodes (Fig. 1a). The transferred graphene film was characterized with Raman spectrum (Fig. 2). The appearance of the three characteristic peaks of the graphene demonstrated the successful transfer of the graphene film onto the glass substrate [24, 25]. The intensity ratio between the 2D band and the G band (I_2D_/I_G_) indicated that the transferred graphene was a multilayer film [26]. Further, the intensity ratio between the D band and the G band (I_D_/I_G_) was small, indicating a very low defect density.

**Fig. 3:**
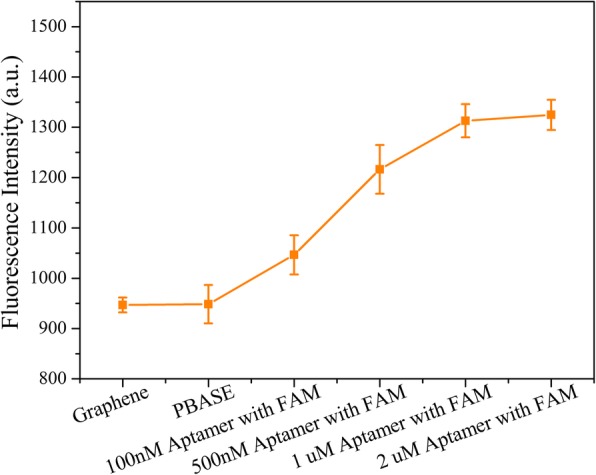
Characterization of MGFETs modification by probe aptamer. Error bar represents the standard deviation of 5 independent analysis

Due to the lack of functional groups, the aptamer chains were difficult to modify on the CVD graphene film. Therefore, based on its aromatic pyrenyl group, PBASE was modified on the graphene films via π–π stacking as a linker. On the other end of PBASE, the succinimide portion of PBASE could be coupled to the 5′-NH_2_-labeled probe aptamer based on the N-hydroxysuccinimide (NHS) crosslinking reaction (Fig. 1c). In order to assess the binding of the probe aptamer on graphene film, the 3′-end of the probe aptamer was labeled using the FAM fluorophore (sequence: 5′-NH_2_-TGG ACC CCC TCA TAA CGC CTC CTT TTC-FAM-3′). Immediately following aptamer introduction, the fluorescence intensity was obviously enhanced, indicating its successful modification on the graphene surface (Fig. 3). Increasing the probe aptamer concentration led to an increase in fluorescence intensity, reaching a constant value, and therefore indicating probe aptamer saturation on the MGFETs, at approximately 2 μM. Therefore, subsequent experiments were performed at a probe aptamer concentration of 2 μM.

### Characterization of MB/cDNA

**Fig. 4:**
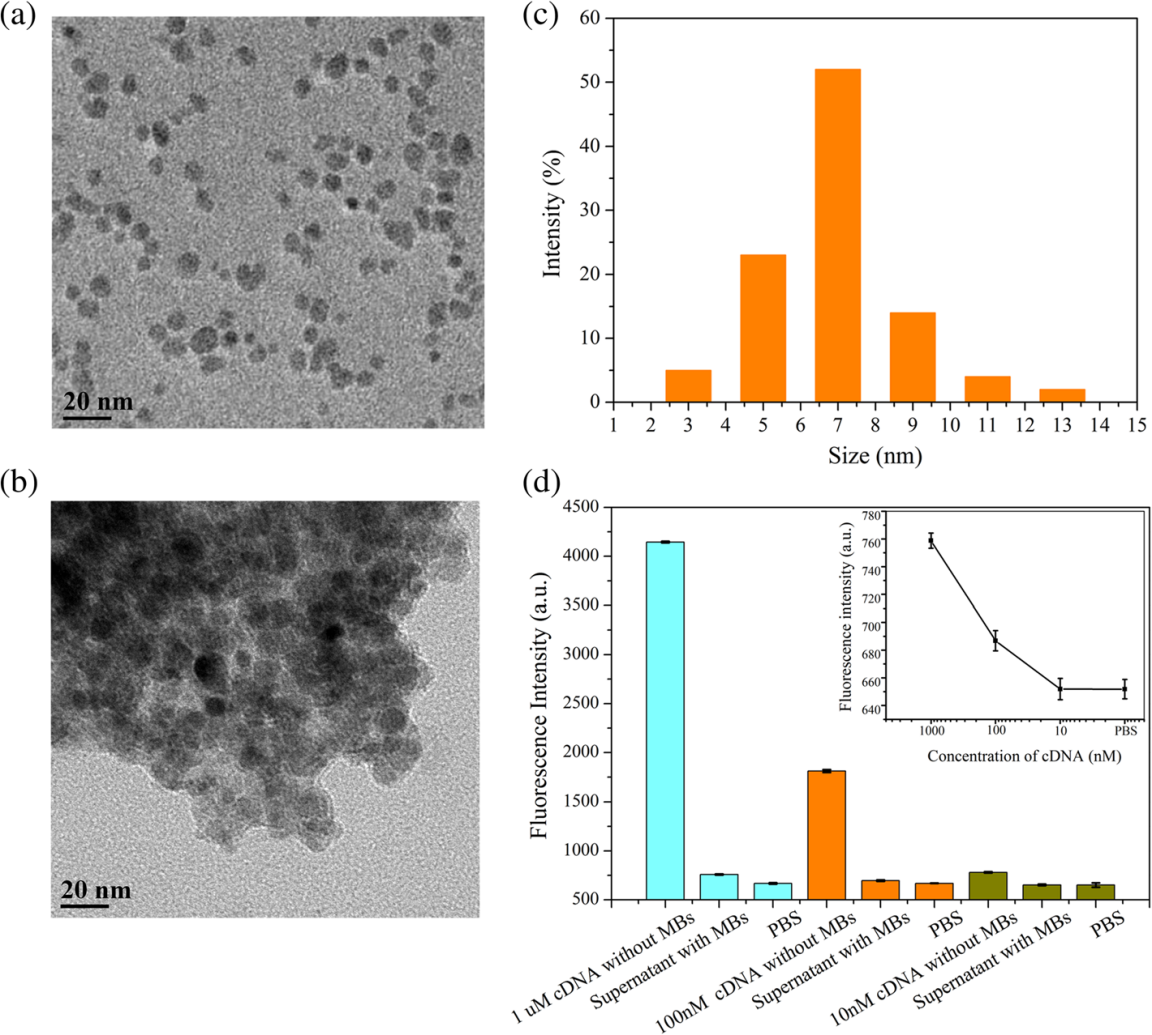
Characterization of MB/cDNA coupling. **a** TEM of MBs. **b** TEM of MB/cDNA conjugates. **c** Particle size distribution of MBs. **d** Characterization of MB/cDNA (FAM) coupling. Error bar represents the standard deviation of 5 independent analysis

The morphology of the MBs and MB/cDNA conjugates was characterized by transmission electron microscopy (TEM) (Fig. 4a, b). The particle size distribution of MBs showed an average particle size of approximately 7 nm (Fig. 4c). In order to ensure sensitivity and accuracy in the biosensing for cDNA, MBs should be excessive for cDNA in order to capture cDNA completely. MBs at a concentration of 4 mg/mL were activated to ensure binding to the cDNA samples use herein. Through labeling of cDNA by FAM, the fluorescence intensity was exploited to characterize the coupling efficiency and optimize the cDNA concentration (Fig. 4d). Indeed, the fluorescence intensity of the supernatant decreased obviously following the introduction of MBs into the cDNA solutions, indicating that cDNA was captured and enriched by the MBs. The successful capturing of cDNA by MBs was confirmed by the observation that, at a cDNA concentration of 10 nM, the fluorescence intensity of the supernatant was equivalent to that of PBS, indicating that all the cDNA was captured and enriched by MBs (Fig. 4d).

### Analysis of Magnetic Field Intensity

MB/cDNA conjugates were added into the MGFETs for 10 min to allow complete cDNA hybridization with the probe aptamer. Since the probe aptamer could not couple with MBs without the modified amino groups, the excess MBs could be removed through washing of the MGFETs three times with PBS. Therefore, only the MB/cDNA conjugates were left on the MGFETs (Fig. 1d). A permanent magnet was mounted onto a rotating motor to apply a periodic magnetic field to the MGFETs (Fig. 1e). A lab-made detection device was used to record the impedance fluctuation of the MGFETs.

**Fig. 5:**
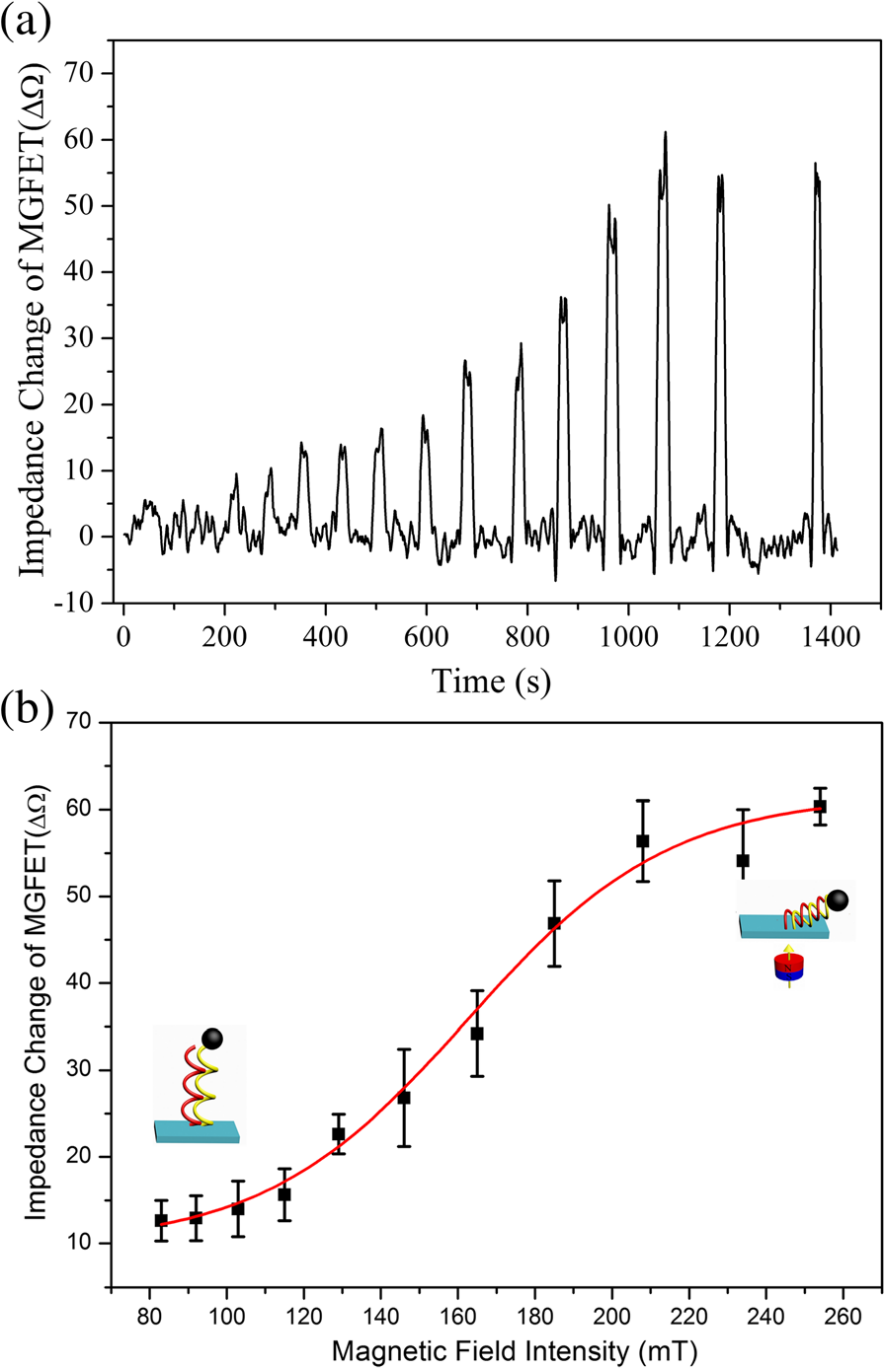
Influence of magnetic field intensity on impedance of MGFETs. **a** Impedance of MGFETs under a varying magnetic field intensity in the time domain. **b** Relationship between impedance of MGFETs and intensity of the magnetic field. Error bar represents the standard deviation of 5 independent analysis

Since impedance of MGFETs was modulated by a magnetic field as the back gate, the correlation between magnetic field intensity and impedance of MGFETs was investigated to optimize the magnetic field intensity parameters (Fig. 5). It is generally believed that the double conductive layer formed between the graphene and the electrolyte is modulated by the external electric field, thereby modulating the conductivity of GFETs [19, 27, 28]. In MGFETs, through the magnetic force between the MBs and the magnetic field, the distance between MB/cDNA conjugates and the graphene film was controlled mechanically, thereby modulating the double conductive layer of MGFETs [29, 30]. MGFET biosensors impedance varied with the increasing magnetic field intensity in three stages which could be explained through taking the MB/cDNA chain as an elastic thin rod [31]. The first stage occurred at a magnetic field intensity of less than 100 mT in this work. Based on the elastic thin rod model of DNA chains, because the magnetic field force is less than the radial support force of the DNA strand, the magnetic field force is difficult to cause the DNA strand to bend; therefore, the MGFETs is not sensitive to the magnetic field. In the second stage with the magnetic field strength from 100 to 200 mT, the magnetic field strength is sufficient to overcome the radial support force of the DNA elastic thin rod, resulting in a rapid bending of the MB/cDNA and then a sensitive response of the MGFETs to the magnetic field. Finally, in the third stage with magnetic field intensity above 220 mT, the bending of the DNA elastic rod reaches its limit; therefore, the MGFETs will not respond to the change of the magnetic field, resulting in a stable impedance of the MGFETs as shown in Fig. 5b.

### Detection of cDNA

The changes in MGFET impedance with varying MB/cDNA conjugate concentrations were measured under a fixed magnetic field strength of 240 mT to determine the feasibility and sensitivity for cDNA detection.

**Fig. 6:**
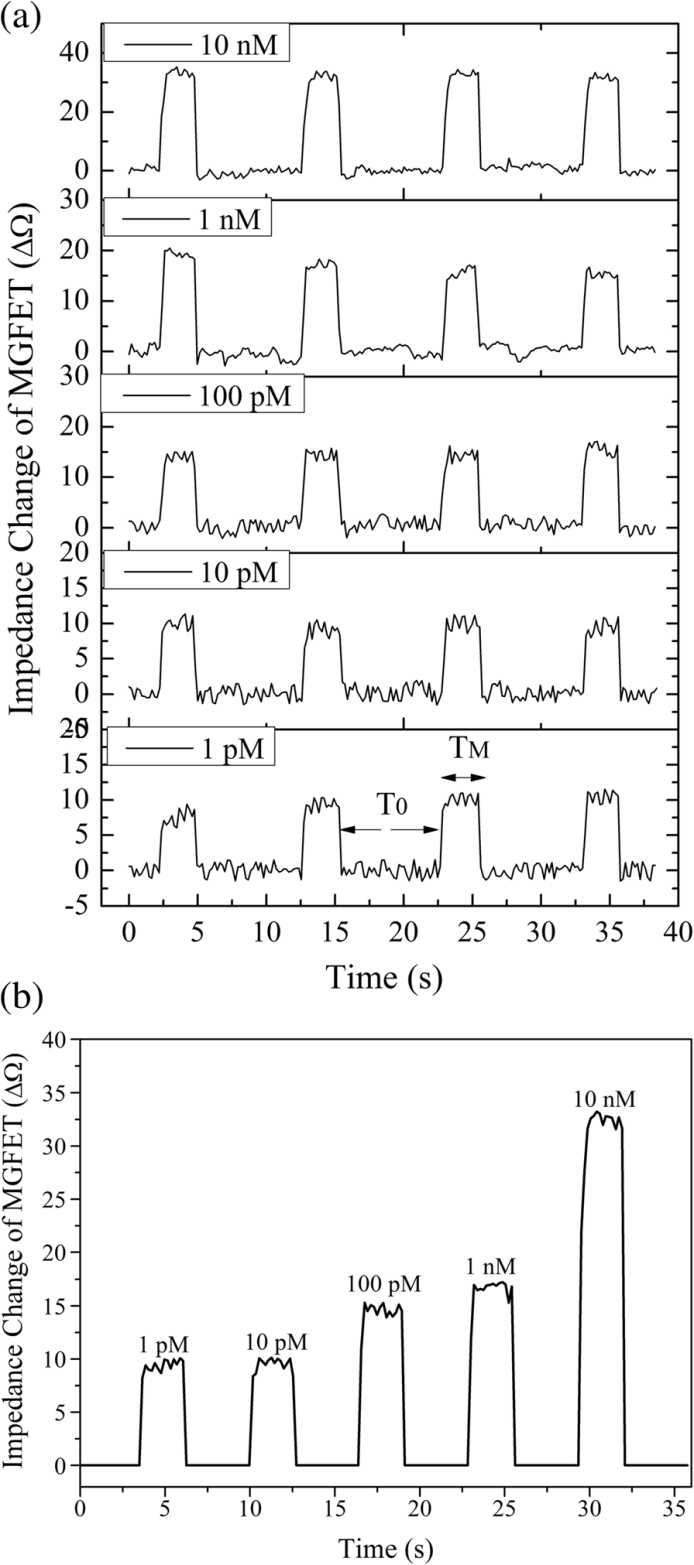
**a** Time domain of impedance fluctuations with different cDNA concentrations. **b** Impedance changes of MGFETs according to cDNA concentration

The MGFET impedance at each cDNA concentration was recorded in real time (Fig. 6a). When a permanent magnet was loaded onto the back of the MGFETs, the impedance increased rapidly. Conversely, when a periodic magnetic field was applied, a periodic change in impedance was observed. Based on this impedance periodicity, a sample integration algorithm (SIA) was used to increase the signal-to-noise ratio of the MGFETs. Given the period without applying magnetic field was T_0_ and the period with applying magnetic field was T_M_ (Fig. 6a), the SIA could be described with the following steps: (1) during T_0_, all the data points, produced by noise, was normalized to zero, (2) the data points obtained during each T_M_ period were sampled and averaged in order. After SIA processing over four cycles, the periodic impedance change in MGFETs was obtained as shown in Fig. 6b. In theory, the signal-to-noise ratio of the MGFETs could be effectively improved using sufficiently long sampling times.

**Fig. 7:**
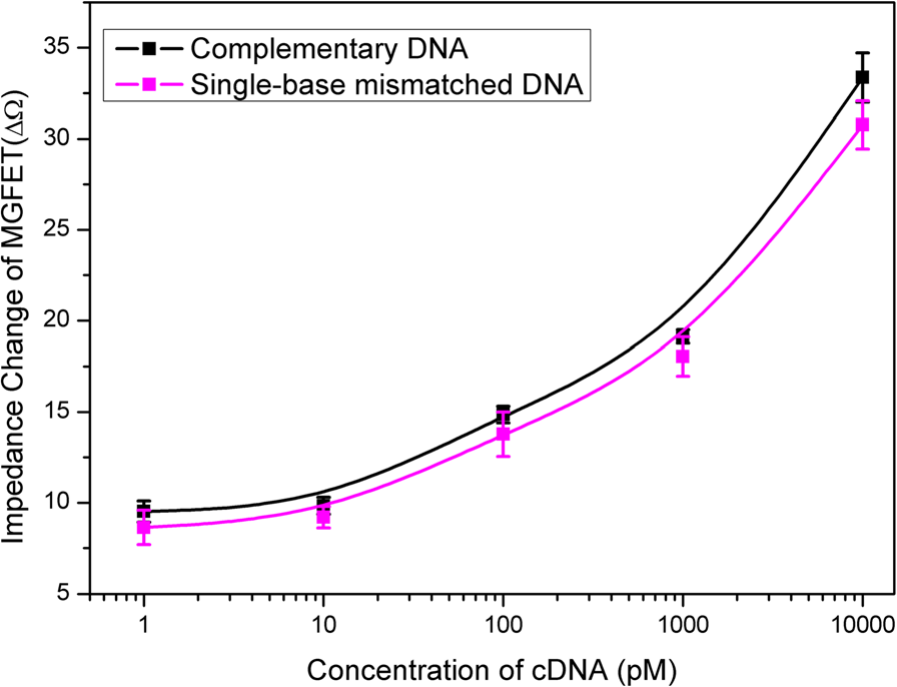
Relationship between impedance of MGFETs and concentration of target DNA. Error bar represents the standard deviation of 5 independent analysis

The impedance changes in MGFETs had a positive correlation with the cDNA concentration (Fig. 6b). The correlation between the impedance change of MGFETs and concentration of cDNA was assessed (Fig. 7). The high sensitivity of the MGFET biosensors in this work is mainly based on the following two aspects: firstly, the mechanical movement of MB/cDNA conjugates could enhance the modulation effect on the double conductive layer compared to the case of DNA alone, and secondly, since a periodic magnetic field could be applied to produce a periodic impedance changes of MGFETs, based on the sampling integration principle, only the MGFET impedance with the magnetic field was sampled and integrated to reduce the noise. Therefore, the system signal-to-noise ratio could be greatly optimized by increasing the number of periods of the external magnetic field.

### Selectivity of the MGFETs

**Fig. 8:**
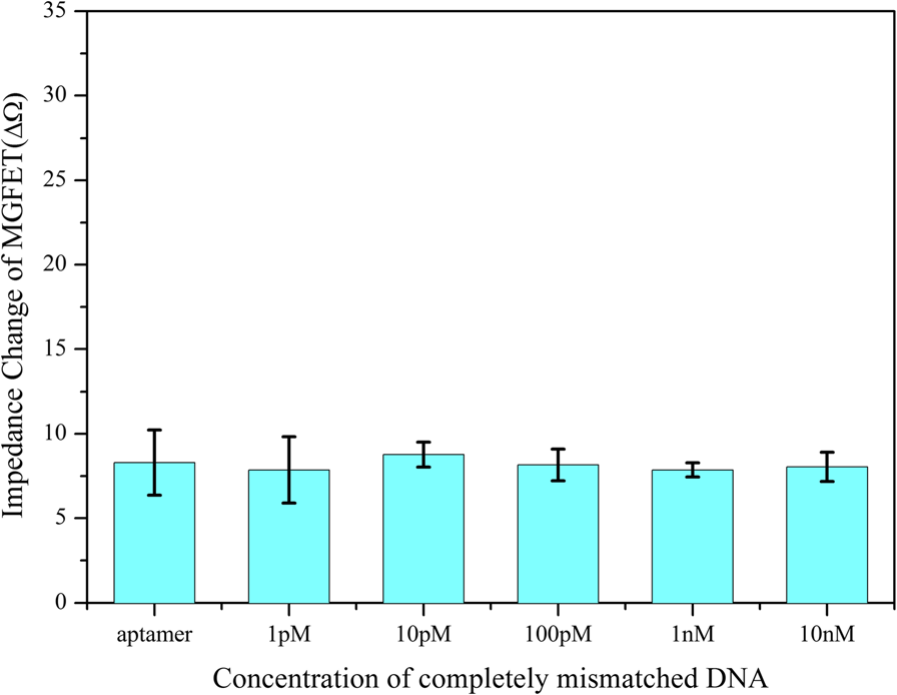
Relationship between impedance of MGFETs and concentration of completely mismatched DNA. Error bar represents the standard deviation of 5 independent analysis

The specificity of the MGFETs was evaluated by detecting two different target DNA sequences, including completely mismatched DNA chains and single-base mismatched DNA chains. Similar to the procedure described above, a completely mismatched DNA (sequence: 5′-NH_2_-TCC CCT TCT TAT GGC CTG TTT TTC AAC-3′) and single-base mismatched DNA (sequence: 5′-NH_2_-GAA AAG GAG TCG TTA TGA GGG GGT CCA-3′) were coupled to MBs respectively. The mismatched MB/DNA dissolved in PBS solution was added into the MGFET biosensors for 10 min to react with the aptamer sufficiently. The MGFETs was washed with PBS for three times to remove the mismatched DNA. For completely mismatched DNA chains, due to the conjugate of MB/DNA could not hybridize with aptamer, almost all the MB/DNA conjugates were removed. Therefore, the addition of completely mismatched MB/DNA has almost no effect on the conductivity of graphene as shown in Fig. 8, which indicates a high selectivity of the biosensor. Furthermore, we have also investigated the selectivity of the biosensors through single-base mismatched DNA chains as shown in Fig. 7. It can be found that the MGFET impedance change with single-base mismatched chains was slightly lower than the complementary strands and higher than the noncomplementary target strand on each certain concentration. Therefore, the single-base mismatched strand could be detectable in this work. Although the aptamer and the complementary DNA chains are all commercial products which mainly determined the selectivity of the biosensors, the MGFETs and its detection system have also provided contribution to the high sensitivity for DNA detection.

## Conclusions

Herein, a MGFET biosensor based on graphene and magnetic nanoparticles was presented to detect cDNA. In the MGFETs, magnetic nanoparticles were modified onto the end of the cDNA sequence. Through the magnetic force between the MBs and the magnetic field, the distance between the MB/cDNA conjugates and the graphene film was mechanically controlled, thereby modulating the double conductive layer of the MGFETs. Furthermore, we can also conclude that, for a particular DNA strand, the impedance of the MGFETs will reflect the stress of the DNA strand, which in turn reflects the bending of the DNA strand (inset, Fig. 5b). Thus, the present MGFETs have the potential to be used in the study of the mechanical parameters of DNA chains. Therefore, the MGFETs may not only function as a biosensor for cDNA detection but may also potentially detect the mechanical parameters of DNA chains.

## Data Availability

All data generated or analyzed during this study are included within the article.

## Abbreviations

cDNA: Complementary magnetically labeled single-stranded DNA
CVD: Chemical vapor deposition
DMSO: Dimethyl sulfoxide
FET: Field-effect transistor
GFET: Graphene field-effect transistor
MBs: Magnetic nanobeads
MGFET: Magnetic graphene field-effect transistor
NHS: N-Hydroxysuccinimide
PBASE: 1-Pyrenebutanoic acid succinimidyl ester
PBS: Sodium dodecyl sulfate phosphate-buffered saline
SDS: Sodium dodecylbenzenesulfonate
SIA: Sample integration algorithm
TEM: Transmission electron microscopy
ITO: Indium tin oxide
